# The Ethics of Cloud Computing

**DOI:** 10.1007/s11948-016-9759-0

**Published:** 2016-02-17

**Authors:** Boudewijn de Bruin, Luciano Floridi

**Affiliations:** 1 Faculty of Philosophy, University of Groningen, Oude Boteringestraat 52, 9712 GL Groningen, The Netherlands; 2 Faculty of Economics and Business, University of Groningen, Nettelbosje 2, 9747 AE Groningen, The Netherlands; 3 Oxford Internet Institute, University of Oxford, 1 St Giles, Oxford, OX1 3JS UK

**Keywords:** Cloud computing, Information ethics, Privacy, Epistemic virtue

## Abstract

Cloud computing is rapidly gaining traction in business. It offers businesses online services on demand (such as Gmail, iCloud and Salesforce) and allows them to cut costs on hardware and IT support. This is the first paper in business ethics dealing with this new technology. It analyzes the informational duties of hosting companies that own and operate cloud computing datacentres (e.g., Amazon). It considers the cloud services providers leasing ‘space in the cloud’ from hosting companies (e.g., Dropbox, Salesforce). And it examines the business and private ‘clouders’ using these services. The first part of the paper argues that hosting companies, services providers and clouders have mutual informational (epistemic) obligations to provide and seek information about relevant issues such as consumer privacy, reliability of services, data mining and data ownership. The concept of interlucency is developed as an epistemic virtue governing ethically effective communication. The second part considers potential forms of government restrictions on or proscriptions against the development and use of cloud computing technology. Referring to the concept of technology neutrality, it argues that interference with hosting companies and cloud services providers is hardly ever necessary or justified. It is argued, too, however, that businesses using cloud services (e.g., banks, law firms, hospitals etc. storing client data in the cloud) will have to follow rather more stringent regulations.

## Introduction

Businesses and individual users alike are embracing online software in order to process, share and synchronize data, recruit personnel, organize customer services and sales, and for an increasing number of other purposes. Computing resources (especially software, memory space, CPU power, and maintenance routines) are becoming services on demand, offered by online providers that store and process files in large datacentres. This new Information Technology (IT) paradigm of *cloud computing* offers huge advantages in terms of installation, configuration, updating, compatibility, costs and computational power (Zhang et al. 2010), and in the last few years cloud computing has already provided enormous benefits to a large number of users. However, it also comes with a number of potential risks. The year 2010, for instance, witnessed a huge cyber attack on the popular cloud email services of Gmail, and the sudden discontinuation of cloud services to WikiLeaks by Amazon. There followed the 2013 NSA spying scandal, the 2014 nude photo iCloud hack and the Sony hack, with hackers increasingly turning to the cloud.

This is the first paper in business ethics dealing with cloud computing.^1^ It employs an informational or epistemic ethical approach (Floridi 2013, 2014a). After a brief overview of cloud computing technology and a survey of the relevant stakeholders, we discuss two issues.

First, we describe the *educational pressure* on *clouders*, that is, initiatives to educate and/or inform the individuals and business corporations that make use of cloud computing services. We observe that cloud computing suits the interests and values of those who adopt a deflated view of the value of ownership and an inflated view of freedom (De Bruin 2010). This is especially, but not exclusively, Generation X or the Millennials, who care less about where, for instance, a certain photograph is stored and who owns it (Facebook? the photographer? the photographed?) and care more about having the opportunity and freedom to do things with it (sharing it with friends, posting it on websites, using it as a background for one’s smartphone). They were aptly described as *Generation Cloud* in a report written by researchers at Goldsmith College, London, and sponsored by Rackspace, a large hosting company.^2^ And they are part of a move towards an Internet of Things in which values shift ‘from the product to the services the product represents’, that is, the Everything-as-a-Service world where one does not need to buy and own, say, a book, but only a licence to read it on one’s Kindle or other device (Melin 2015). We use insights gained from the epistemic study of freedom (De Bruin 2010) to argue that this warrants particular forms of *educational pressure*. Not only must the clouders discharge their epistemic duties. The cloud computing industry must also develop radically different ways to communicate with its customers. The industry should have concern for the virtue of *interlucency*, as we call it (De Bruin 2015). It should communicate with customers, provide them with information about what the technology does, and actively check whether these customers understand what it attempts to communicate.

Second, we describe the *proscriptive pressure* on the cloud computing industry and its *business* customers, that is, proscriptions about what they must not do. Our point of departure is that the companies hosting cloud services and providing the hardware, the infrastructure and platform (Amazon, Google, Microsoft and several other firms) as well as the companies providing cloud services, the applications and the software (Salesforce, ZoHo Recruit, Google Docs and many others) should receive as little proscriptive pressure as possible. The motivation that underlies this assumption is that, on the one hand, regulation of this emerging industry would run the risk of stifling innovation, while, on the other hand, as far as we can see now the risks of cloud computing technology are much less significant than, say, the risks of nuclear power or genetic engineering. The largest risks of cloud computing concern its use and misuse, and hence we suggest that proscriptive pressure must be exerted primarily on the business *users* of software as a service rather than on the *providers*.

Cloud computing is still in its infancy. Most of the research literature comes from computer science and concerns the technology (see, e.g., Erl et al. 2013; Zhang et al. 2010). Many business magazines have devoted attention to the reasons for adopting cloud computing in business, to questions about the financial performance of specific companies, and, to a lesser extent, to the possible dangers (Palmer and Bradshaw 2011). There is also a significant literature in law, sketching, for instance, the consequences for cloud computing of proposals for legal reform in the EU (Hon et al. 2014).

The present paper differs from these lines of literature. Unlike the computers science and business literature, it approaches cloud computing at a fairly general and non-technical level; and unlike the law literature, we consider not so much the specific legal mechanisms through which cloud computing can be regulated, but rather approach regulation from an ethical rather than legal point of view, which, to our knowledge, is novel.

Our main position can be summed up as follows: we encourage the cloud computing industry and its business customers to take a step forward yet cautiously, making haste slowly, as the Latin adage *festina lente* suggests. At the same time, our aim with this paper is to convince the business ethics community of the relevance of studying cloud computing and the many ethical issues surrounding it, in order to anticipate problems that, tackled earlier, are more easily solvable.

## Cloud Computing

The history of the word *cloud computing* goes back at least as far as a description of the ATM networks that became widespread during the 1990s. But it was not until 2006, when Eric Schmidt, Google’s CEO at the time, started using the term, that it became popular in its current meaning. Right now it is being used in more than twenty different ways (Vaquero et al. 2009) and is just as much exploited by marketing departments as it is met with scepticism from anti-Internet sides (Zhang et al. 2010; Moreno-Vozmediano et al. 2013). It has a common core, though, which we shall introduce in a non-technical way here below.^3^

We write this paper using a word processing program that runs on our laptops. We use software installed on these laptops; we use the laptops’ processors to run the software; and we use the laptops’ hard drives to store versions of the paper. But we could have written it using the online suite offered by Google Docs. If we had done that, we would have used software installed on computers in Google’s datacentres, scattered around the world; we would have used Google’s processors and servers to run the software; we would have used their computers to store only one file. We would have been working in the *cloud*. And where the way we actually wrote the paper required sending versions of it back and forth by email, Google Docs would have allowed us to ‘share’ the document and to work on one and the same file in the cloud, even simultaneously.

Cloud computing does not just make it easier for people to work on joint projects. More than that, it decreases the need for businesses to invest in fancy computers, data servers, expensive software that you only use once a month, maintenance and support staff, and many other things. What you need is a rather plain computer, connected to the Internet, some basic software, like a free browser and a pay-as-you-go subscription to the services that you need. The servers in the datacentres may go down of course. To minimize the risk of interrupted service due to power outages, datacentres are located near power plants and data are stored on various different physical locations—the greater the number of locations where your data are stored, the more you pay. Google, for instance, has datacentres in Oklahoma, Oregon and a few other US states, as well as in Belgium, Chile, Finland, Ireland, The Netherlands, Singapore and Changhua County, Taiwan.^4^ This is also true for other companies. Yet even then, things may go wrong. Cloud services may face problems as a result of which they become temporarily unavailable. For the numerous companies dependent on cloud services, this means interruption of their websites, their customer services and/or their sales administrations. Small start-up companies are typically affected most: cloud companies require their customers to pay more to store data in more datacentres to diminish the risk, but smaller companies are less likely to be able to afford this. Insurance companies have started developing products covering some of the risks of power outage and service interruption, marketing them both to providers and customers of cloud services, but it is unclear to what extent smaller companies benefit from this development.

Cloud computing is not a new idea. Its business model goes back to the old days of computing, when companies and researchers rented computation time on large mainframe computers. These developments were pioneered by such firms as IBM in the 1950s, and ideas of computation as a utility function—which cloud computing, like water and electricity, really is—were further championed in the 1960s by John McCarthy, the Stanford computer scientist and Artificial Intelligence pioneer, and by Douglas Parkhill, then Canada’s Assistant Deputy Minister for Research (McCarthy 1961; Parkhill 1966).

The Personal Computer (PC) changed this idea of public utility—only temporarily, of course, if cloud computing will live up to its promises. Public utility was abandoned in favour of an ideal of computation as a private affair, carried out at home, in the office, in isolation, with the explicit suggestion that this would ‘liberate’ the computer user. It surely did. The main reason that the PC gave users increasingly more freedom was, however, not that the physical location of the computation changed, but rather that PCs would become increasingly powerful. And while outsourcing computation to the cloud makes computation a less private and stand-alone business, and more like a public utility again, cloud computing represents still another increase in computational power. Some authors describe the change as just as radical as the one invoked by the PC (Carr 2008), and indeed, the most popular email providers worldwide, Hotmail and Gmail, are cloud services.

It is useful to distinguish between a number of actors in and around the cloud computing industry. First, there are *hosting companies* that own and run the datacentres, the servers, the hard disks on which the data are stored and the processors necessary for the computation. Examples include Amazon, Google, Microsoft and Rackspace.^5^ Then, there are the *cloud services providers*, which provide specific online services. These services are sometimes called Software-as-a-Service (SaaS) and examples include Google Docs (word processing, spreadsheet, etc.), Salesforce (customers services and sales), ZoHo Recruit (recruitment), Dropbox (sharing and synchronizing data) and many others.^6^ And third, there are the *clouders*, the individual or business customers of service providers that use SaaS at home or in business. Every Google Docs user is a *clouder*. Business examples are companies using Salesforce to manage their sales administration, using the cloud-based survey tools of SurveyMonkey for market research, or using online tools to store customer accounts in the cloud. These *business clouders* are an interesting category. They do not, strictly speaking, provide cloud services; they use them. The customers of business clouders, however, may not always spot the difference between a cloud services provider and a business clouder using Software-as-a-Service, or may fail to realize that, when they fill in data on online customer loyalty websites, they are in reality contributing data to the cloud.

## Stakeholders

An informational ethical approach to cloud computing starts with an identification of the stakeholders that are affected by cloud computing (Floridi 2013; Zakhem et al. 2007). Stakeholders are individuals or groups that are confronted with the consequences of corporate activities, affecting their interests or rights. They typically include owners, investors, employers, employees, customers, suppliers, competitors, governments and the environment (Freeman 1984). Who are they? What are their rights, their interests, their vulnerabilities? What possible advantages and disadvantages may cloud computing services have for them?

To start with the last one, the *environment* is an easily forgotten stakeholder. Datacentres consume large amounts of energy; about half of the energy goes to cooling the processors only. A 2010 study by Pike Research suggested that, in comparison to a business as usual scenario, the adoption of cloud computing may well reduce energy consumption by almost 40 % (Pike Research 2010). The largest gain here, it was argued, is obtained by outsourcing computational tasks from inefficient local datacentres (or home and office computers) to the more efficient large datacentres of the hosting companies. Environmental advantages are also to be expected because cloud computing decreases the need to invest in ever more powerful hardware because data are saved and computational tasks carried out by servers and processors in the cloud (Berl et al. 2010). Yet recent publications caution against overly rash and optimistic scenarios (Mastelic et al. 2015).

*Governments*, in turn, are stakeholders in two ways. First of all, governments have to respond to new technology by developing new laws or not. But governments can also assume the function of hosting company, cloud services provider or business clouder. The Dutch government, for instance, has decided not to experiment with cloud computing services available from private vendors and has therefore developed its own ‘closed’ cloud for its own IT functions. The City of San Francisco, on the other hand, has been a pioneer in moving services into the commercial, ‘open’ cloud for some time (Walton 2011). And the UK government attempts to steer the middle course between open and closed clouds by setting up the ‘G-Cloud framework’, which is a rather lightly regulated marketplace where the cloud computing industry and the public sector meet.

Next we turn to *investors* in cloud computing companies and the cloud computing industry. Reliable figures are hard to find, but analysts at UBS, the Swiss investment bank, estimate that revenues from Amazon Web Services, the cloud division of Amazon, was only around $200 million in 2010; by the third quarter of 2015 it had grown to a staggering $2.1 billion. Some cloud computing companies do not have the goal of contributing to technological innovation and offer relatively simple filing, storage or backup services (e.g., Dropbox). These firms typically buy the services of larger companies that invest heavily in the design and building of faster and increasingly efficient datacentres (Amazon), while still others are mainly concerned with the development of cloud computing software (Salesforce). This last category, the cloud services providers, boosts impressive results, too.

Following the distinction between private and business clouders, cloud computing has advantages and disadvantages for both, but not all of them are the same. Cloud computing decreases the need for installation, configuration and updating of software, but does not reduce it to zero—one needs to set up and configure an account, for instance. For larger businesses this may lead to a significant reduction of costs, because traditionally software had to be installed, configured and maintained on every single desktop in the office. For smaller businesses and private clouders, the change may be less significant. Yet even if costs do not decrease, there may be positive effects on cash flow due to the fact that cloud services providers use a pay-as-you-go pricing system. Disadvantages vary as well. Private clouders and small start-up companies, for instance, may not be willing (or capable) to pay the full rate and have to content themselves with free or low-cost services that are accompanied by pop-up ads, limited downloading and uploading, less than maximal reliability and other drawbacks. Secondly, when they pay more, the functionality of the service may become too complex, designed as it often is with the interests of large business clouders in mind, rather than those of private users or small business clouders. Continuous payment of fees may be required for keeping your data safe, particularly when clouders do not have the resources for backup storage themselves. Moreover, when software changes, data formats may change, with uncertainty about backward compatibility.

A loose category of stakeholders includes those *individuals*, business *corporations* and others whose data are stored in the cloud, not by themselves, but by individuals or businesses with whom they interact. This happens when a business collects information about its customers, and stores and processes it by means of such applications as Salesforce, but also when patients or doctors store medical files in the cloud, or even simpler when a customer sends an email to a business Gmail account.

A final category includes stakeholders that are indirectly affected by cloud computing. A few years ago, a study by the Brookings Institution claimed that a large part of the savings that cloud computing promises are due to reducing IT jobs, in particular IT support staff (West 2011), but in all fairness it should be noted that the debate about potential negative effects of cloud computing on employment has waned. A more serious worry today concerns citizens in developing countries, where even the more optimistic scenarios still allow for the possibility that cloud computing may exacerbate rather then diminish the digital divide between developed and developing countries (Floridi 2007). While cloud computing seems to be a boon to a population that cannot afford the computer equipment that is necessary for today’s IT—a very simple laptop is sufficient for cloud computing—it also requires reliable, ubiquitous and high speed Internet connections that are almost entirely absent, and if not absent very expensive, in large parts of the world.

## Educational Pressure

In order to get a clear grasp of what normative requirements follow from our observations so far, we turn to recent changes in the views held by clouders about ownership and freedom. The main idea is that many users of IT services have gradually adopted a *deflated* view of the value of *ownership*, a sense that owning things is no longer as important as it was (De Bruin 2010). This is particularly true of Generation Y, the Millennials, the generation born in the eighties and nineties (Howe and Strauss 2000). This generation has large expertise with electronic devices and electronic commerce, is concerned with the community, oriented towards teamwork, and it attaches great value to ‘sharing’ things. One aspect of this is that Generation Y accepts the rules they learned from their parents to a greater extent than Generation X (born in the sixties and seventies). Where parents and educators are absent, though, Generation Y follows their own rules; and these rules often reveal a deflated view of ownership, reflected in a more lenient or perhaps simply different attitude towards plagiarism and Internet piracy (Freestone and Mittchel 2004). Information available on the Internet is not seen as belonging to someone whose property rights have to be respected; rather, it is seen as something put out there to be shared and to be freely used (Germek 2009).

While a deflated view of ownership is most clearly visible in Generation Y, this generation is by no means unique in this respect. A significant proportion of the stakeholders affected by cloud computing embrace such a view. And it is this view of ownership, we shall argue now, that motivates the need for specific educational pressure on clouders. First, we defend the view that a deflated view of ownership often entails an inflated view of freedom. Then, we interpret this using an epistemic view of arguments for the value of freedom (De Bruin 2010). This enables us, finally, to defend our claim about the need for educational pressure on clouders, indicating the epistemic responsibilities both of the cloud computing industry (and those businesses using their services) as well as of the clouders themselves.

With a deflated view of the value of ownership, it is no longer ownership that counts, but the use that people can make of a certain thing. We move from owners to users, who do not so much value possessing a certain hard copy of a photograph, but rather the fact that they can view photographs, show them to their friends, include them on their homepage and in their social network profiles, or manipulate them in Photoshop. They value ownership only instrumentally insofar as it gives them opportunities, that is, freedom of choice. Oversimplifying: ownership that yields no freedom loses its value.

A standard argument for the value of freedom is to the effect that freedom allows people to satisfy their desires, to fulfil their wishes and to reach their goals (Carter 1995; Kreps 1979; De Bruin 2010). If your freedom increases, the likelihood increases that among the actions you are free to select there is an action that would satisfy your desires best. Another argument goes back to Kant and focuses on personal responsibility, maintaining that if your freedom increases, your responsibility increases too, because you are responsible for *excluding* increasingly more options (Hurka 1987). In this sense, an increase in freedom is an increase in opportunity costs (Benn 1975). These arguments are often invoked by politicians and policy makers to motivate specific forms of regulation (Brown 2009). However, as (De Bruin 2010) has shown, the value of freedom is best realized when specific epistemic conditions are satisfied, which politicians and policy makers tend to overlook. To benefit genuinely from their freedom, people have to know what actions they can choose from and they have to know what the likely consequences of these various choice options are. In other words, they have to know the characteristics of their opportunities.

These observations form the basis of a number of responsibilities that cloud computing stakeholders must assume. If cloud computing is to deliver on its promise to cater to the desires of people embracing an inflated view of freedom of choice, then realizing the conditions necessary for freedom of choice to be exploited ought to be given priority. It is here, then, that educational pressure on the clouders enters the stage, both for private and business clouders. Clouders need to have general knowledge about the advantages and disadvantages of cloud computing; and they need to have specific knowledge about the services they buy and use or consider buying or using.

Information about the advantages and disadvantages of cloud computing will primarily have to be provided by the hosting companies and the cloud services providers, because they have the most extensive and up to date knowledge. The typical ways by which companies communicate with their customers are advertisements (commercials) and licence agreements, however, and none of these are particularly adequate to get a good view of one’s options needed for an informed decision on the part of the customer. Commercials do of course emphasize the advantages, but sometimes exaggerate them and rarely mention the disadvantages. Licence agreements do mention the risks and disadvantages, but they are not a very good source of information either, because they are written in hard to understand ‘legalese’, which causes customers not to read the texts of the licence agreements and only check the required ‘consent boxes’ in order to obtain access to site or service.

Now it surely cannot all hang on the cloud computing industry and their business customers only. The industry is not the ‘clouder’s keeper’ (Ebejer and Morden 1988). Clouders, too, have to do some epistemic work. In particular, they have to search for information. Recent work on virtue epistemology is useful to flesh out the responsibilities of the clouders with a little bit more precision. Authors such as Montmarquet (1993) and Zagzebski (1996) have developed rather sophisticated theories of epistemic virtues that, like the non-epistemic or practical virtues, give normative guidance to individual human behaviour, an approach that is gaining traction in applied ethics as well (Crossman and Doshi 2014; Marcum 2008; Rawwas et al. 2013; De Bruin 2013).^7^ *Intellectual impartiality* is one of these epistemic virtues. Intellectually impartial clouders consider cloud computing in an open-minded way and are willing to confront their prejudices (about alleged insecurity or data mining, for instance) with opposing ideas, while being actively aware of the fact that their own beliefs might be wrong. They listen to what the cloud computing industry says, but they will also actively seek recommendations from independent experts and representatives of consumer organizations. Another epistemic virtue is *intellectual sobriety*. Intellectually sober clouders resist the overly enthusiastic adoption of beliefs about either the pros or the cons of cloud computing; they take ads with the necessary grain of salt. At the same time, they avoid being overly sceptical, because scepticism leads to inaction. They realize that making a business decision forces them to make up their mind and to decide what to believe, for instance, when they must decide on whether to buy new locally installed software or subscribe to cloud computing services. The third virtue is *intellectual courage*. Intellectually courageous clouders admit their own ignorance and keep actively searching for information if they need it, even if they meet resistance—or even contempt—from others. If they do not understand the terms of service, they will ask; and if they do not understand the answer, they will ask again.

Yet even the most epistemically virtuous clouder will fail to collect sufficient information to make an informed decision if hosting companies, cloud services providers and business clouders do not communicate in the right way. Terms of use are often cast in very lengthy documents written in a legal jargon many people find hard to understand. This is one of the main barriers obstructing adequate communication between the industry and its customers. O’Neill (2011) has diagnosed this as a form of ‘quasi-communication’ that primarily serves the function of laying off liabilities rather than ensuring that clouders understand what services they buy into. The solution we suggest here is that the cloud computing industry should strive for *interlucent* communication (De Bruin 2015). *Interlucency* is an epistemic virtue. Yet unlike the virtues of intellectual impartiality, sobriety and courage, which are self-regarding virtues, interlucency has to do with the way agents interact with other agents; it is an other-regarding or patient-oriented epistemic virtue (Kawall 2002; De Bruin 2015). Interlucency incorporates the virtues of being a good teacher. Interlucent agents make sure to adapt the provision of information to the audience they want to reach, and they actively track whether their audience is understanding them.

Slightly more formally, interlucency can be seen as an epistemic virtue directed at establishing *common knowledge* to the extent that this is necessary for successful communication. A proposition φ is common knowledge among two agents A and B whenever both know that φ is true, both know of each other that they know that φ is true, both know that both know that φ is true, and so on. Common knowledge captures situations in which φ is completely open and transparent to the relevant agents. In game theory, common knowledge is seen as a source of beliefs that agents need for coordinated action and social cooperation (Geanakoplos 1992; De Bruin 2005). Linguists have used these insights to understand communication and mutual understanding between speakers and hearers, and to show that a breakdown of common knowledge about the meaning of a certain linguistic utterance is likely to result in miscommunication. Suppose, for instance, that A tells B to get the book from a library. Normally it will be common knowledge between A and B that *library* refers to an institution where you can borrow books. But this may clearly be upset by B’s knowing that A is French and that in French *librairie* means bookshop rather than library.

As a communicative ideal, common knowledge implies such things as that speakers use words not just in ways that are correct according to the dictionary; what should also guide their linguistic choice is whether what they say is likely to be understood by the hearers in the intended way. It is here that interlucency comes into play. If an agent A has evidence to the effect that φ, and A knows that φ has to be communicated to B, then A will use communication strategies that B is likely to interpret correctly. And this often requires more than just sending the message. It also requires checking whether B has understood the message, and if not, to find alternative ways to communicate φ. To that end, A has to examine what background information B possesses, what, for instance, the level of technicality is that B will understand or whether B will give common words like *anonymous* or *personal data* the precise legal meaning A may give to them.

Interlucency is somewhat related to Habermas’ (1973) concept of *Verständlichkeit* ‘comprehensibility’, which may be seen as a precondition of communicative action. While comprehensibility does not get as much attention in Habermas’ (1981) own writings as the better known concepts of truth, rightness and truthfulness, it has found its way in applied contexts inside (Porr 2005; Spahn 2012) and outside philosophy (O’Donnell and Henriksen 2002; Underwood and Ozanne 1998). Comprehensibility is, however, more general than interlucency in the sense that it captures the syntactic and formal aspects that communication action should satisfy. Interlucency, by contrast, is always related to specific speakers and hearers and the specific epistemic demands that they have to satisfy for communication between them to be successful.

Regulation may force businesses to be interlucent. In the UK and other countries, for instance, buyers of certain financial services have to go through a lengthy, detailed *and clear* presentation of the risks of the products they buy, and they have to sign a form indicating that the risks have been explained to them in full. Certain mortgage products cannot be bought without the consumer having demonstrated a clear understanding of how they work. These procedures contribute to the establishment of common knowledge among clients and service providers. Other ways to implement interlucency are lists of Frequently Asked Questions—if indeed these are the questions that are frequently asked—or accurate query-answering services by email or in discussion forums.

Similar measures have much to recommend themselves to the cloud computing industry, and there is reason to assume that they may work. Dropbox, one of the most popular cloud file synchronization services used by academics and business people alike, stated in an earlier version of its licence agreement (in 2010) that ‘By utilizing the site…you consent to allow Dropbox access to your computer to access any files that are placed in the…folder you choose to link to Dropbox’. This left many questions unanswered. Did this mean that storing a file in such a folder entailed giving Dropbox staff access to it so that they could *read* it? That would have meant that Dropbox could engage in data mining of what you store on the site. Nowhere in the 2010 agreement did Dropbox clarify this issue, even though at the time it was one of the most serious concerns clouders had about cloud computing services (Fujitsu 2010). Dropbox answers the data mining question in a forum: ‘if you’re really paranoid you can monitor all network communication of Dropbox, but let me just say up front that you shouldn’t be putting anything like medical records (which plenty of people have inquired about) into Dropbox for legal reasons’.^8^ Today, however, Dropbox has a fairly elaborate and easy to navigate section devoted to privacy issues, answering many potential concerns of their customers.^9^

One may wonder how our recommendation to increase interlucency in cloud computing through regulation can be squared with proposals to reform EU data protection law. One of the main pillars of existing data protection law is the *notice and consent* model of consumer informed consent. A number of commentators think, however, that this model is outdated because in the age of Big Data ICT makes it possible to analyse large amounts of data gathered from a large variety of different sources in ways that cannot be described to consenting consumers in understandable ways or that simply cannot be predicted beforehand (Mantelero 2014). A suggested solution is the establishment of data protection authorities that, endowed with sufficient technological knowledge and expertise, shall speak on behalf of the consumers.

It cannot be sensibly denied that a number of technical questions are too complex for most consumers to address. It is also true that, as the notice and consent framework is actually implemented, it is often too easy for companies ‘to give notice and require the consent without effective self-determination of users’ (Mantelero 2014), that is, failing to establish genuine informed consent. Moreover, data processing increasingly targets not only individual people but also social groups (ethnic or religious groups, local communities, nations, etc.), which shows the importance of a concern for group privacy (Floridi 2014b). Yet there will remain numerous issues in which the notice and consent model is far from outdated. Many of the more tangible risks that consumers of cloud computing run can be described to them in ways that they understand. Not disputing the potential relevance of data protection impact assessments and other initiatives meant to keep a tab on the processing of personal data, we do not believe that the notice and consent model can be set aside so easily. Even when potential future use of data is hard to predict, the primary guiding normative principle ought to be that customers must be in the position to decide for themselves how to deal with the existing uncertainty rather than outsourcing their decisions to data protection authorities.

This is not to say that the notice and consent model as we know it should be left unchanged. We agree with the critics that the current implementation of the model does not always succeed in generating genuine informed consent. We propose that this is often due not to the fact that by its very nature the required information is too complex to understand for most customers, but rather to the fact that it was not communicated by the service provider in a way that customers understand. It is here that we see the potential contributions of interlucency come to the fore most clearly, because it suggests a more context-dependent approach to duties of information and transparency. Merely providing information in transparent ways is not enough for communication to be interlucent. Interlucent service providers tailor their communications to their intended audience, and track their understanding, because they realise that genuine informed consent requires first and foremost that the consumers understand the information on which their consent depends. In our view, current as well as proposed regulation too often allows the industry to obtain consent through forms that are too complex for most consumers to understand. Our suggestion here is that regulation should require the industry not only to provide information, but to provide it in ways that consumers understand, and that the industry must actively check whether customers understand.

## Proscriptive Pressure

Recall our distinction between *hosting companies* owning and operating the datacentres; *cloud services providers* developing particular forms of Software-as-a-Service and leasing ‘space in the cloud’ from hosting companies; and *business clouders* that use these cloud computing services. We work from the assumption that minimal proscriptive pressure must be put on hosting companies and cloud services providers, but that rather extensive proscriptive pressure may be exerted on business clouders. This assumption gains plausibility from a broadly liberal principle connecting freedom and technological progress. It is based on the idea that even though scientific and technological developments may have disadvantages, governments (and other regulators) will hardly be able to predict the disadvantageous outcomes of research and development and that they should therefore minimize interference during the development phase. This argument can be found in the writings of such authors as John Stuart Mill (1859) and Friedrich von Hayek (1960), but it has been defended with more precision by Carter (1995). The claim is not that developing clearly harmful technology should be allowed; it does not readily apply to nuclear power, say, the risks of which are rather straightforward to determine. Rather, the idea is that in a situation in which clear indications of serious downside risks are so far lacking, government bans are premature. From this perspective, the cloud computing industry requires only minor proscriptive pressure. Of course this is restricted to the initial stages of product development, because downside risks may become visible along the way; and if that happens, government policy may have to be re-evaluated.

Another defence of this assumption refers to the chilling effects that regulation may have. Regulation may force IT businesses into specific directions and even have negative spill-over effects in other domains (Reed 2007), when, out of fear for legal repercussions, companies stay on the safe side and develop products only if there is no doubt that they are legally acceptable. This would stifle creativity and innovation. A government may, for instance, require the cloud computing industry to satisfy certain standards of security or reliability, or prohibit data mining or marketing through personalized ads, and it may do so with the intention to protect consumers. But at the same time, the argument goes, such regulation may make cloud computing more expensive to customers who, for instance, may not need the extra 0.99 % reliability or security, or to those who have good reasons not to object to data mining.

Our claim that minimal prospective pressure ought to be exerted on hosting companies and cloud services providers—but sometimes rather intense pressure on business clouders—is in line with demands for *technology neutral* regulation. Our defence does not discriminate against particular technologies. Nor does not hamper the development of technology. Rather, treating business clouders differently from hosting companies and cloud services providers rests on a conception of technology neutrality to the effect that the purpose of regulation is to regulate effects, not means (Knoops 2006). As we shall show shortly, it is in particular the effects of the activities of certain business clouders that are ethically problematic.

It is true that regulation of business clouders may have chilling effects, too, and that certain (non-ICT) businesses may for fear of non-compliance decide against adopting cloud computing technology that, if they adopted it, would lead to efficiency improvements. But we do not think these risks will likely materialize. And even if they do, this will not so much impact the development of ICT but rather put a break on efficiency enlarging measures in those businesses that can very well operate without cloud computing. Businesses that make essential use of cloud computing will take the risk.

This does not let the cloud computing industry off the hook. As we argued in the previous section, the other side of the coin is that the cloud computing industry has an obligation to communicate in crystal-clear fashion with their consumers. If the idea is that the industry (hosting companies and cloud services providers) and its customers are left free more or less to do and contract what they think is to their mutual advantage (which is what we argued for in the first paragraphs of this section), then consumers must have detailed and adequate knowledge of what they actually buy. And as we have seen, this requires more than merely finessing detailed licence agreements; it requires the kind of genuine interlucent communication that we defended in the previous section.

As we indicated before, while we argue for limited proscriptive pressure on hosting companies and cloud services providers, proscriptive pressure on businesses making use of cloud services, the *business clouders*, will have to be considerably stronger. Here, too, the guiding principle is that regulation should not stifle innovation, but since the main activities of business clouders is something else than cloud computing, proscriptive pressure is less likely to have such an effect. To argue in favour of proscriptive pressure on business clouders, we shall now discuss a number of properties of cloud computing that, through the activities of business clouders, may negatively affect certain stakeholders. We shall also indicate what proscriptive pressure may be used as a response.

To begin with, the physical security of datacentres themselves determines the likelihood of servers, and therefore data, being stolen. Even though online crime is more common, criminals have shown some interest in actual servers, and several legal cases show that the data stored on these servers were used for criminal purposes (De Bruin 2010). Yet ultimately the probability of this kind of crime is likely to decrease when firms start opting for cloud services, because criminals will find it very hard to determine which servers in the datacentres contain the data they are interested in. Whereas a bank’s server has only one purpose and is an easy target for criminals interested in data on social security numbers, credit card numbers and the like, cloud computing datacentres store very different kinds of data and this may makes it less attractive to burgle a cloud computing datacentre. Because of their larger impact, physical terrorist attacks on datacentres were expected in the first years of cloud computing. However, a more serious concern seems to be the use terrorist groups make of cloud computing services themselves, as well as ‘non-physical’ cyber attacks on datacentres, including the 2014 Sony hack. And while physical attacks on datacentres can be prevented by traditional methods, hosting companies are in constant competition with cyber criminals honing their decryption and hacking skills.

Sometimes such skills are not even needed to gain access to certain data. In the bulk of cases, the physical location where the data are stored determines the jurisdiction under which it falls. Once data cross national boundaries, it may be much easier for interested parties to gain access even in legal ways. Law enforcement in the US and elsewhere increasingly contacts hosting companies and cloud services providers with requests to make customer data available. In the first 6 months of 2014, the number of data requests received by Google from US law enforcement agencies amounted to 12,539, of which 84 % were completely or partially complied with.^10^ It cannot be denied that the search warrants that underlie some of these requests may play a crucial role in law enforcement, and we believe that there are cases where cloud search warrants are fully justified. All the same, what these figures show is also that the cloud is not a safe place for a particular kind of data. Lawyers, for instance, must be forbidden to store some kinds of customer data in the cloud. Here we strongly disagree with the ethics opinions issued by several professional organizations according to which decisions about storing customer data have to be left to the lawyer’s discretion (Acello 2010). Despite the fact that other voices can be heard defending more stringent codes of conduct (Lewallen 2013), these opinions are still fairly common. But while they are right to point to the advantages of using cloud computing in general and to play down the risk of cyber attacks and other security breaches, they seriously ignore the fact that when, say, a European lawyer stores data in the cloud and the data end up in a datacentre in the US, the data may fall under US law, with unforeseen consequences—and there is no guarantee either that hosting companies will not extend their territory to countries with poor or no legislation protecting customers.^11^ Not to mention the risk of technical failures where, as Lewallen (2013) describes, legal documents that a law firm had stored on Google Docs were forwarded to all people with whom it had shared documents in the past.

Interested parties can exercise influence on the cloud in other ways as well. In a widely publicized event in the history of cloud computing, the staff of Joe Liberman, Chairman of the US Senate’s Homeland Security and Governmental Affairs Committee, contacted Amazon apparently with the request to remove WikiLeaks from its servers. A day later, the hosting company indeed discontinued their service to WikiLeaks. In a dry comment, the *Guardian* wrote that this is a ‘wake-up call to anyone who thinks that Cloud Computing services can be trusted to protect the interests of customers’ (Naughton 2011).

There is then a third reason why security breaches are likely to increase concerns about the way in which business clouders and private clouders access the cloud. Cloud services such as Salesforce are very attractive for business people working at many different locations, because they can access their customers’ data from the office computer, but also while travelling, using their laptop, or from home. To log on to these cloud services, one typically needs a username and a password, but for convenience many users save them on their computers so that they are automatically logged on to the cloud services when they start their computers. If these computers are not themselves protected (by passwords or fingerprints, for instance), anyone who gains access to the computer has access to the cloud services and hence to the data of numerous customers. Moreover, public wifi networks at airports, conference venues and so on are likely to be a prominent form of access to the Internet for many business people, in particular if they are working for smaller companies that cannot afford more expensive mobile Internet. The security of these networks is, however, far from optimal. If business people turn to the cloud and start storing sensitive customer data there, such data breach cases will increase—even if the number of stolen laptops remains the same—because the cloud services will contain more data than can be stored on one laptop alone.

## Conclusion

Increasingly more private and business customers are turning to the cloud as the default option. The advantages are indeed huge: no installation, no configuration, no updating, no upgrading, no compatibility problems, low costs, and computation power that far exceeds that of their own computers, their own servers and their own datacentres. This is very attractive to many business corporations that have witnessed a data explosion (so called Big Data) that their in-house computing resources can no longer handle. Banks, pharmaceutical industries, insurance companies, marketing, consultancy and research firms, and many others benefit enormously whenever the cloud computing industry makes highly complex computer tasks possible and affordable, by combining innumerable processors spread all over the world. However, there are risks to cloud computing, too. First of all, many clouders are unaware of what cloud computing really amounts to. We have argued that this is due to a lack of interlucent communication between the cloud computing industry and its customers, and showed that competing with integrity in this emerging market requires of hosting companies and cloud services providers that they do their utmost to ensure that customers understand what they buy. Second, we defended the claim that regulation of the hosting companies and the cloud services providers should be at a minimum, because proscriptive pressure here risks slowing down innovation. Yet regulation of the business customers of the cloud services providers is urgently needed. Hosting companies and cloud services providers move their customers’ data with high frequency from one datacentre to another so as to enable efficient use of storage space. This is one of the innovations that marks cloud computing. But it is currently unsuitable, we have argued, to store lawyers’ client data, for instance, or sensitive military, business or medical data. Disagreeing with several professional associations, we defended, for instance, the claim that lawyers should be forbidden to store client data in the cloud.

To our knowledge, this is the first paper dealing with cloud computing from the perspective of business ethics. The technology is still in its infancy, and while computer scientists have of course amply published on the topic, its ethical implications have been largely ignored so far. This has made some of the conclusions of this paper tentative, depending as they do on a relatively slim body of research. We hope that this paper may inspire other researchers to take up this fascinating subject.

## Footnotes

1 Other papers on ethics and cloud computing are Greenbaum and Gerstein (2011), on privacy and genetic data, Ratten (2012), on the psychology of ethical decision making and cloud computing, Stark and Tierney (2014), on privacy and Lockbox, an encrypted cloud application, and Timmermans et al. (2010), on general conceptual issues surrounding cloud computing.

2
http://www.rackspace.co.uk/sites/default/files/whitepapers/generation_cloud.pdf (accessed 30 March 2015).

3 The definition suggested by the National Institute of Standards and Technology seems to find relatively wide acceptance among scholars See http://csrc.nist.gov/publications/nistpubs/800-145/SP800-145.pdf for the 16th and final draft (accessed 30 March 2015).

4
http://www.google/corporate/datacenter/locations.html (accessed 30 March 2015).

5 Technically, individuals or businesses may host their own cloud server through network-attached storage devices such as MyCloud (Western Digital) or CloudBox (Lacie), facilitating backups and access. These products do not offer Software-as-a-Service, which makes them less interesting for businesses.

6 Hosting companies typically offer cloud services as well, witness Google Docs and Microsoft Office 365.

7 The exposition here is based primarily on Montmarquet (1993).

8
http://forums.dropbox/topic.php?id=7833 (accessed 29 June 2011).

9
https://www.dropbox.com/help/topics/security_and_privacy (accessed 30 March 2015).

10 See http://www.google.com/transparencyreport/userdatarequests/ (accessed 30 March 2015, data for second half of 2014 not yet available). The country ranking second is Germany with 3338 requests in the same period of which 48 % were complied with.

11 This is among the reasons underlying EU sponsorship of a Europe-wide cloud computing scheme.

## References

[CR1] Acello R (2010). Get your head in the cloud. ABA Journal.

[CR3] Benn S (1975). Freedom, autonomy, and the concept of a person. Proceedings of the Aristotelian Society New Series.

[CR4] Berl A, Gelenbe E, Di Girolamo M, Giuliani G, De Meer H, Dang MQ, Pentikousis K (2010). Energy-efficient cloud computing. The Computer Journal.

[CR6] Brown A (2009). Personal responsibility: Why it matters.

[CR7] Carr N (2008). The big switch.

[CR8] Carter I (1995). The independent value of freedom. Ethics.

[CR10] Crossman J, Doshi V (2014). When *not knowing* is a virtue: A business ethics perspective. Journal of Business Ethics.

[CR11] de Bruin B (2005). Game theory in philosophy. Topoi.

[CR12] de Bruin B (2010). The liberal value of privacy. Law and Philosophy.

[CR13] de Bruin B (2013). Epistemic virtues in business. Journal of Business Ethics.

[CR14] de Bruin B (2015). Ethics and the global financial crisis: Why incompetence is worse than greed.

[CR17] Ebejer J, Morden M (1988). Paternalism in the marketplace: Should a salesman be his buyer’s keeper?. Journal of Business Ethics.

[CR18] Erl T, Mahmood Z, Puttini R (2013). Cloud computing: Concepts, technology and architecture.

[CR20] Floridi L (2007). Global information ethics: The importance of being environmentally earnest. International Journal of Technology and Human Interaction.

[CR21] Floridi L (2013). The ethics of information.

[CR22] Floridi L (2014). The fourth revolution: How the infosphere is reshaping human reality.

[CR23] Floridi L (2014). Open data, data protection, and group privacy. Philosophy & Technology.

[CR24] Freeman RE (1984). Strategic management: A stakeholder approach.

[CR25] Freestone O, Mittchel V-W (2004). Generation Y attitudes towards e-ethics and internet-related misbehaviours. Journal of Business Ethics.

[CR26] Fujitsu. (2010). *Personal data in the cloud: A survey of consumer attitudes*. Fujitsu Limited.

[CR78] Geanakoplos J (1992). Common knowledge. Journal of Economic Perspectives.

[CR27] Germek G (2009). Imagine no possessions: Librarians, the net-generation student and the imminent victory of plagiarism. College & Undergraduate Libraries.

[CR29] Greenbaum D, Gerstein M (2011). The role of cloud computing in managing the deluge of potentially private genetic data. American Journal of Bioethics.

[CR31] Habermas J, Fahrenbach H (1973). Wahrheitstheorien. Wirklichkeit und Reflexion.

[CR32] Habermas J (1981). Theorie des kommunikativen Handelns.

[CR34] Hon, W., Kosta, E., Millard, C., & Stefanatou, S. (2014). *Cloud accountability: The likely impact of the proposed EU data protection regulation*. Tilburg Law School Legal Studies Research Paper Series No. 07/2014.

[CR35] Howe N, Strauss W (2000). Millennials rising: The next great generation.

[CR36] Hurka T (1987). Why value autonomy?. Social Theory and Practice.

[CR37] Kawall J (2002). Other-regarding epistemic virtues. Ratio.

[CR38] Knoops B-J, Knoops B-J, Lips M, Prins C, Schellekens M (2006). Should ICT regulation be technology-neutral?. Starting points for ICT regulation: Deconstructing prevalent policy one-liners.

[CR39] Kreps D (1979). A representation theorem for ‘preference for flexibility. Econometrica.

[CR40] Lewallen M (2013). Cloud computing: A lawyer’s ethical duty to act with reasonable care when storing client confidences ‘in the cloud’. Cleveland State Law Review.

[CR43] Mantelero A (2014). The future of consumer data protection in the E.U.: Re-thinking the ‘notice and consent’ paradigm in the new era of predictive analytics. Computer Law and Security Review.

[CR44] Marcum J (2008). The epistemically virtuous clinician. Theoretical Medicine and Bioethics.

[CR45] Mastelic T,  Oleksiak A, Claussen H, Brandic I, Pierson JM, Vasilakos AV (2008). Cloud computing: Survey on energy efficiency. ACM Computer Surveys.

[CR46] McCarthy J (1961). Centennial Keynote address.

[CR48] Melin H (2015). Consumer empowerment in the Internet of Things: A silent unfolding of a ‘new normal’ where code trumps rights?. International In-house Counsel Journal.

[CR49] Mill JS (1859). On liberty.

[CR50] Montmarquet J (1993). Epistemic virtue and doxastic responsibility.

[CR51] Moreno-Vozmediano R, Montero R, Llorente I (2013). Key challenges in cloud computing: Enabling the future internet of service. Internet computing: IEEE.

[CR52] Naughton, J. (2011, February 6). How Twitter engineers outwitted Mubarak in one weekend. *Guardian.*

[CR54] O’Donnell D, Henriksen L (2002). Philosophical foundations for a critical evaluation of the social impact of ICT. Journal of Information Technology.

[CR55] O’Neill, O. (2011). *Trust and mediated communication*. Paper presented at ‘The Philosophy of Trust and Cloud Computing’, Cambridge.

[CR56] Palmer, M., & Bradshaw, T. (2011). Storm of publicity for cloud computing. *Financial Times*.

[CR57] Parkhill D (1966). The challenge of computer utility.

[CR59] Pike Research. (2010). Cloud computing energy efficiency: Strategic and tactical assessment of energy savings and carbon emissions reduction opportunities for data centers utilizing SaaS, IaaS and PaaS.

[CR61] Porr C (2005). Shifting from preconceptions to pure wonderment. Nursing Philosophy.

[CR62] Ratten V (2012). Entrepreneurial and ethical adoption behaviour of cloud computing. Journal of High Technology Management Review.

[CR63] Rawwas M, Arjoon S, Sidani Y (2013). An introduction of epistemology to business ethics: A study of marketing middle-managers. Journal of Business Ethics.

[CR64] Reed C (2007). Taking sides on technology neutrality. SCRIPT-ed.

[CR67] Spahn A (2012). And lead us (not) into persuasion…? Persuasive technology and the ethics of communication. Science and Engineering Ethics.

[CR68] Stark L, Tierney M (2014). Lockbox: Mobility, privacy, and values in cloud storage. Journal of Business Ethics.

[CR69] Timmermans J, Stahl B, Ikonen V, Bozdag E (2010). The ethics of cloud computing: A conceptual review. IEEE Second International Conference on Cloud Computing Technology and Science.

[CR70] Underwood R, Ozanne J (1998). Is your package an effective communicator? A normative framework for increasing the communicative competence of packaging. Journal of Marketing Communications.

[CR71] Vaquero L, Rodero-Merino L, Caceres J, Lindner M (2009). A break in the clouds: Towards a cloud definition. ACM SIGCOMM Computer Communications Review.

[CR72] von Hayek F (1960). The constitution of liberty.

[CR73] Walton, J. (2011). *How the cloud helps government agencies deliver more to their constituents*. Paper presented at ‘CloudSlam’11: Cloud Computing Virtual Conference’.

[CR74] West D (2011). Saving money through cloud computing.

[CR75] Zagzebski L (1996). Virtues of the mind: An inquiry into the nature of virtue and the ethical foundations of knowledge.

[CR76] Zakhem A, Palmer D, Stoll M (2007). Stakeholder theory: Essential readings in ethical leadership and management.

[CR77] Zhang Q, Cheng L, Boutaba R (2010). Cloud computing: State-of-the-art and research challenges’. Journal of Internet Service Applications.

